# Development and validation of a nomogram for predicting bone metastasis in breast cancer: a retrospective study

**DOI:** 10.3389/fsurg.2025.1722983

**Published:** 2026-01-20

**Authors:** Yingnan Li, Teng Ma, Xinyi Sun, Changgen Liu, Haibo Wang

**Affiliations:** Breast Disease Diagnosis and Treatment Center, The Affiliated Hospital of Qingdao University, Qingdao, China

**Keywords:** bone metastasis, breast cancer, cancer, nomogram, predictive model

## Abstract

**Background:**

Bone metastasis is the most common site of distant metastasis in breast cancer. Patients with bone metastasis have their quality of life and survival rate threatened. This study aims to develop a practical nomogram for predicting the risk of bone metastasis in breast cancer by integrating clinical data, assisting doctors in making more scientific clinical decisions.

**Methods:**

We conducted a retrospective analysis of the data of newly diagnosed breast cancer patients from the database of the Affiliated Hospital of Qingdao University from January 2015 to December 2017. The cohort is divided into training set and validation set in a ratio of 7.5:2.5. Determine independent risk factors through Least Absolute Shrinkage and Selection Operator (LASSO) regression analysis and logistic regression, and develop a nomogram prediction model. The model's performance and clinical utility were evaluated by Receiver Operating Characteristic (ROC) curve analysis, Area Under the Curve (AUC), calibration curves, and Decision Curve Analysis (DCA).

**Results:**

During the 5-year follow-up period, bone metastases developed in 48 of 421 patients (11.40%). Ultimately, six independent risk factors were identified: neoadjuvant chemotherapy, family history of cancer, distant metastasis in other locations, axillary lymph node metastasis, marital status, and primary tumor site. The nomogram demonstrated excellent predictive performance, with AUC values of 0.89 and 0.86 in the training and validation cohorts, respectively.

**Conclusions:**

This pioneering nomogram, incorporating baseline, tumor characteristics, and therapeutic parameters, provides visual guidance for breast surgeons to assess bone metastasis risk in breast cancer patients. It enables clinicians to prioritize high-risk patients through early identification, thereby optimizing surveillance protocols and therapeutic strategies to safeguard patients' quality of life.

## 1 Introduction

Breast cancer, characterized by its distinctive epidemiological patterns and marked heterogeneity, remains one of the leading causes of cancer-related mortality among women worldwide. According to global statistics, over 2 million new cases are diagnosed annually, substantially compromising both the quality of life and survival outcomes for affected individuals (1). Reportedly, the 5-year overall survival rate exceeds 80% in patients with non-metastatic breast cancer, whereas the development of distant metastasis drastically reduces the 5-year survival rate to approximately 25% (2). bone metastasis accounts for over 75% of distant metastases in breast cancer, constituting the most frequent site of distant spread (3). Bone metastasis in breast cancer can induce skeletal-related events (SREs), including pain, functional impairment, hypercalcemia, pathological fractures, spinal cord and nerve root compression, and bone marrow infiltration, contributing to diminished quality of life in affected patients (4, 5). Patients suffering from bone metastases tend to demonstrate an extended overall survival (OS) in comparison to individuals with metastases affecting visceral organs. However, the presence of intense bone pain can severely affect their functional abilities. As a result, this leads to a considerable decline in their quality of life (6).

Current research indicates that the risk factors for bone metastasis in breast cancer remain controversial. This topic has been extensively investigated in both domestic and international studies. Numerous clinical investigations have established that factors such as the patient's age, tumor grade, molecular subtype, histological classification, along with the existence of concurrent metastases in the lung, liver, or brain, serve as independent risk determinants for bone metastasis in breast cancer (7–10). Furthermore, human epidermal growth factor receptor 2 (HER2) positivity has been identified as a risk factor for both primary and metastatic breast cancer (11). However, emerging research paradoxically suggests that human epidermal growth factor receptor 2 (HER2) positivity may act as a protective factor in metastatic breast cancer (12). The Tumor-Node-Metastasis (TNM) staging system is widely employed in clinical decision-making and prognosis prediction. However, it falls short in adequately quantifying and predicting tumor biology and the risk of distant metastasis (13, 14). Recent studies have constructed various predictive tools using machine learning, such as risk scores derived from the Surveillance, Epidemiology, and End Results (SEER) database and predictive models incorporating hematological parameters, facilitating preliminary risk predictions (7, 9). However, current models may exhibit considerable heterogeneity in the weighting of risk factors. For instance, the impact of hormone receptor status on risk shows inconsistent directions across different studies (10, 15). Current research may still fail to achieve accurate prediction of breast cancer bone metastasis. Moreover, individualized prediction is susceptible to misclassification. Therefore, we aim to construct a novel nomogram model. This model seeks to address these critical constraints.

Additionally, radiation exposure from diagnostic imaging is a frequent concern in clinical practice (16). Although nuclear medicine examinations, such as bone scans, effectively detect bone metastasis, their utility is often limited. This limitation stems from public apprehension and financial constraints (17). Therefore, the development of a straightforward nomogram to effectively predict malignant bone metastasis risk carries significant clinical implications.

Compared to traditional scoring systems, nomograms offer visual and personalized advantages. A nomogram graphically represents regression equations. It simplifies outcome prediction by integrating continuous and discrete variables through interactive axes (18). Unlike single-factor-based risk stratification, nomograms employ multivariate analysis. They integrate weighted factors for comprehensive assessment (19). This provides a visual approach to accurately predict individual bone metastasis risk in breast cancer patients. Consequently, it facilitates optimization of personalized treatment plans. Most current breast cancer bone metastasis risk prediction models are derived from public databases. Their applicability to Asian populations remains unclear (7, 20). Note that studies on breast cancer bone metastasis prediction models based on region-specific data remain scarce.

Consequently, the objective of this research is to create and validate a nomogram designed for the personalized assessment of the risk of bone metastasis in patients diagnosed with breast cancer. From comprehensively collected demographic and clinical data, we built a nomogram integrating these multifaceted features. The nomogram estimates the likelihood of bone metastasis in breast cancer patients, aiding clinicians in evidence-based therapeutic decision-making.

## 2 Patients and methods

### 2.1 Research design

**Figure 1:**
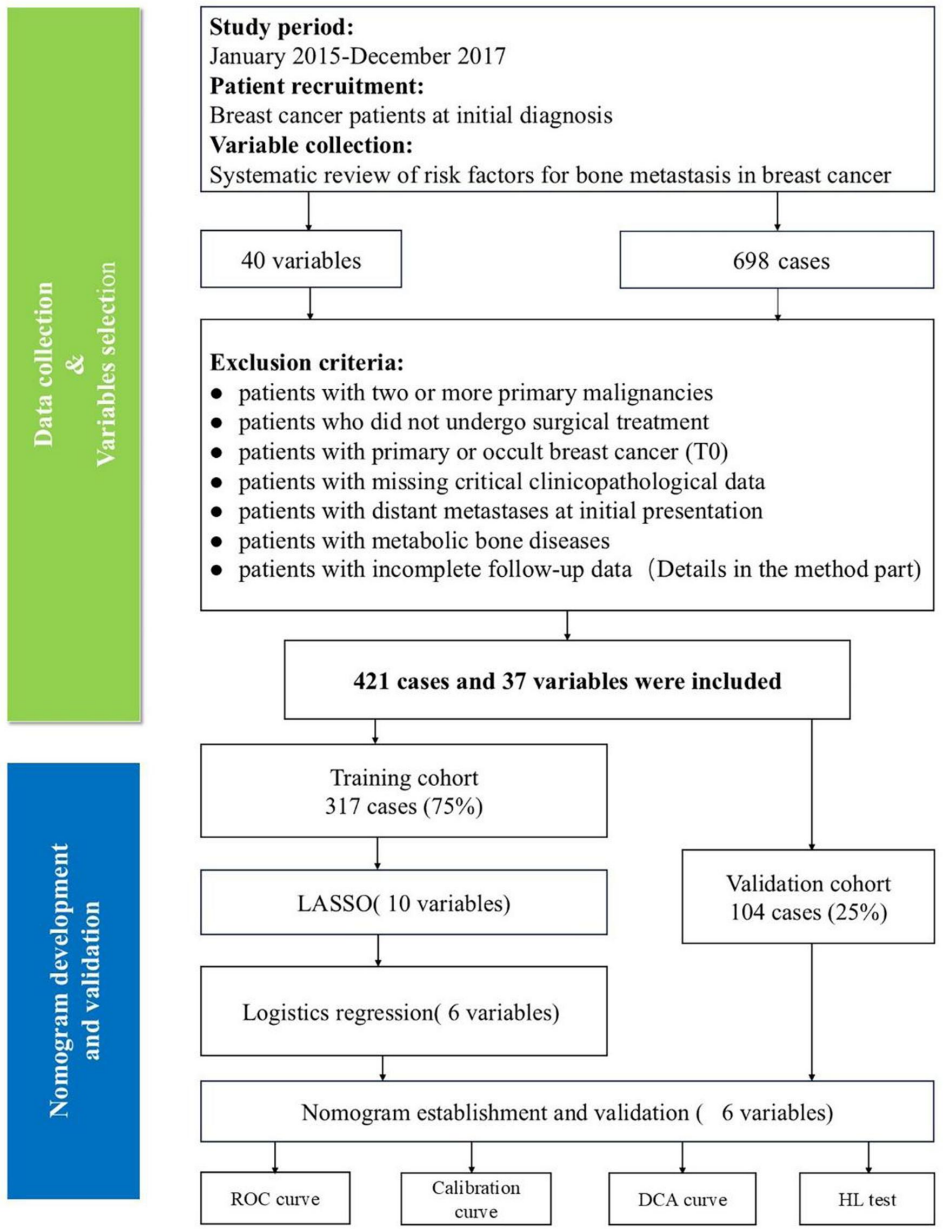
Study flowchart. ROC, receiver operating characteristic; DCA, decision curve analysis; HL, Hosmer-Lemeshow.

This single-center retrospective study utilized anonymized clinical data from this center. A flow diagram presents the overall study design, including patient screening and variable collection processes (Figure 1).

### 2.2 Patient information

This study was approved by the Ethics Committee of The Affiliated Hospital of Qingdao University. We included patients newly diagnosed with breast cancer between January 2015 and December 2017; these patients were subsequently followed for 60 months. All baseline and clinical data were retrieved from the MediCloud Database at The Affiliated Hospital of Qingdao University (ethics approval number: QYFY WZLL 29986, retrospectively registered). Informed consent, which was documented in writing, was secured from all patients participating in the study at the time of their admission. All methodologies in this study strictly adhered to the principles of the Declaration of Helsinki.

### 2.3 Inclusion and exclusion criteria

Patients meeting the following criteria were enrolled: (1) Histopathologically confirmed primary breast cancer; (2) Primary tumor staged as T1 (maximum tumor diameter ≤2 cm), T2 (maximum tumor diameter >2 cm and ≤5 cm), or T3 (maximum tumor diameter >5 cm), with complete TNM stage documentation (21); (3) Clearly documented interval from initial breast cancer diagnosis to bone metastasis development; (4) Complete baseline clinicopathological data, including histological grade, hormone receptor (ER/PR) status, HER2 status, Ki-67 index, and molecular subtype; (5) Traceable comprehensive treatment records, including surgical approach, neoadjuvant/adjuvant chemotherapy, radiotherapy, and endocrine therapy regimens; (6) Age ≥18 years with regular follow-up data.

Exclusion criteria were: (1) Male breast cancer patients; (2) Patients with ≥2 primary malignancies; (3) Patients not undergoing surgical treatment; (4) Patients with primary occult breast carcinoma (T0); (5) Patients lacking essential clinicopathological information, including primary tumor location, TNM stage (22), estrogen receptor (ER) and progesterone receptor (PR) status, HER2 status, histological type, and axillary lymph node metastasis (ALNM) status; (6) Patients presenting with distant metastasis at initial diagnosis; (7) Patients with concurrent bone metabolic diseases; (8) Patients lost to follow-up or whose death was unrelated to breast cancer during follow-up (e.g., accidents or other severe illnesses).

### 2.4 Data collection and determination of main results

All variables included in this study were categorized into baseline characteristics, tumor stage, pathological information, diagnostic testing data, and clinical treatment information (Table 1). Laboratory parameters reflect the first hematological tests obtained upon admission.

**Table 1:** Baseline data for training and validation sets.

Variables	Training group	Validation group	*p* values
	(*n* = 317)	(*n* = 104)	
Demographic and basic information			
Age	49.98 ± 10.54	49.00 ± 11.45	0.42
Age at Menarche	15.04 ± 1.67	15.01 ± 1.56	0.88
BMI	24.74 ± 3.37	24.71 ± 2.89	0.94
Height	160.96 ± 5.92	160.49 ± 4.50	0.46
Menopause (No/Yes)	120/197	43/61	0.60
Literality (Left/Right)	169/148	53/51	0.76
Family History of Cancer (No/Yes)	245/72	80/24	1.00
Marital status			
(Unmarried/Married/Widowed/Divorced)	5/296/14/2	2/97/3/2	0.60
Abortion			
(No/Once/Twice/Three or more times)	148/81/58/30	40/37/19/8	0.23
Insurance			
(FullSelf-payment/UrbanEmployee Medical Insurance/UrbanResident Medical Insurance/Others)	44/175/70/28	14/56/25/9	0.98
Blood type (O/A/B/AB)	87/90/99/41	27/35/28/14	0.73
Laboratory Parameters			
Hb	128.44 ± 14.06	129.65 ± 15.22	0.45
Ca²⁺	2.25 ± 0.12	2.27 ± 0.12	0.58
ALP	61.44 ± 19.30	60.87 ± 21.53	0.80
CEA	1.80 ± 2.29	1.64 ± 1.46	0.43
CA125	14.93 ± 10.50	17.39 ± 19.08	0.95
CA153	11.30 ± 13.46	10.88 ± 6.99	0.76
Ki67	33.22 ± 21.42	33.90 ± 21.43	0.78
Cancer Characteristics			
Histological types			
(InvasiveDuctalCarcinoma/Invasive Lobular Carcinoma/Others)	289/10/18	101/1/2	0.13
Grade (I/II/III)	30/178/109	8/63/33	0.70
Tumor count (1/2/3)	282/26/9	93/9/2	0.87
Primary site			
(Upper-outer quadrant of breast/Lower-outer quadrant of breast/Upper-inner quadrant of breast/Lower-inner quadrant of breast/Accumulated nipple)	167/46/67/32/5	48/15/31/8/2	0.45
T Stage (T1/T2/T3)	184/124/9	53/48/3	N/A
N Stage (N0/N1/N2/N3)	184/77/28/28	58/27/7/12	N/A
ALNM (No/Less than 4/At least 4)	198/66/53	60/24/20	0.68
Distant Metastasis in Other Locations			
No Additional Distant Metastases/Lung Metastasis/Lung Metastasis/Liver Metastasis/Brain Metastasis	285/17/7/8	89/6/4/5	0.51
Breast subtype			
(HR+/HER2-/HR+/HER2+/HR+/HER-2+/HR/HER2−)	198/44/38/37	65/14/13/12	1.00
ER (Negative/Positive)	78/239	26/78	1.00
PR (Negative/Positive)	102/215	34/70	1.00
HER2 Status (Negative/Positive)	234/83	77/27	1.00
Targeted Therapy (No/Yes)	257/60	85/19	0.99
Treatment Information			
Neoadjuvant Chemotherapy (No/Yes)	290/27	93/11	N/A
Targeted Therapy (No/Yes)	257/60	85/19	N/A
Endocrine Therapy (No/Yes)	82/235	28/76	N/A
Radiotherapy (No/Yes)	199/118	55/49	N/A
Chemotherapy (No/Yes)	83/234	19/85	N/A
Surgery			
(SimpleMastectom/RadicalSurgery/Breast-conserving Surgery/Total Reconstruction after Resection)	77/151/66/23	19/56/21/8	N/A

*p*-values for T stage, N stage and treatment variables are not applicable (N/A) as these are not randomly assigned characteristics but are determined by disease presentation and standard clinical guidelines.

Diagnosis of bone metastasis required fulfillment of at least one criterion: (1) Detection of metastatic breast cancer cells in bone biopsy; (2) Positive findings on bone scintigraphy and/or positron emission tomography (PET)/PET-CT scan, with confirmation by CT, MRI, or radiography when necessary, demonstrating imaging characteristics consistent with typical bone metastasis (e.g., osteolytic/blastic destruction on CT/MRI) (23). All diagnostic procedures were performed within affiliated departments of our institution.

### 2.5 Sample size calculation

The sample size for this study was estimated *a priori* based on the Events Per Variable (EPV) criterion, a well-established method for developing multivariable prediction models to ensure reliable parameter estimates and minimize overfitting.

The expected event rate (p) for the primary outcome bone metastasis, was conservatively set at 15%. This estimate was derived from preliminary analysis of our institutional data.

We anticipated that the final logistic regression model would retain between 4 and 6 key predictor variables following clinical and statistical selection. To ensure a robust sample size, we used the upper limit of this range (6 variables) and the standard minimum EPV value of 10 for calculation.

The minimum sample size was calculated in two steps:

- The required number of events (*E*) was determined as: *E* = (Number of predictor variables) × (*EPV*) = 6 × 10 = 60 events.
- The total minimum sample size (*N*) was then derived using the formula: *N* = *E*/*p* = 60/0.15 = 400 participants.

This calculation indicated that a minimum of 400 patients was required to develop a stable model. Our final analyzed cohort comprised 421 patients, which exceeds this requirement and provides adequate statistical power for the objectives of this study.

### 2.6 Establishing and evaluating a nomogram prediction model

This study utilized R software (version 4.4.2) to develop the nomogram prediction model and generate assessment plots. To select the most relevant predictors from a high-dimensional dataset and prevent overfitting, we performed LASSO (Least Absolute Shrinkage and Selection Operator) regression using the “glmnet” package. The variables selected by LASSO were then entered into a multivariable logistic regression model (fitted with the “glm” function) to establish the final prediction model and estimate the regression coefficients (24).

The “rms” package was employed to generate the nomogram (a graphical tool for calculating individual risk) and calibration curve (which plots predicted probabilities against observed frequencies to assess accuracy). We utilized the “rmda” package to conduct decision curve analysis (Decision Curve Analysis, DCA) for assessing the net benefit of our prediction model. The model's discriminative ability was assessed by constructing a receiver operating characteristic (ROC) curve and calculating the area under the curve (AUC) using the “pROC” package.

Ultimately, model performance was comprehensively evaluated from three perspectives: discrimination (AUC), calibration (calibration curve), and clinical utility (DCA).

### 2.7 Statistical analysis

Continuous variables are expressed as mean ± SD; categorical variables as counts (*n*). Continuous variables were compared with the Student's t-test; categorical variables with the *χ*² test or Fisher's exact test. Statistical significance was defined as *p* < 0.05.

## 3 Results

### 3.1 Patient demographics

This study enrolled 421 breast cancer patients with a mean age of 49.98 ± 10.54 years. In the 5 years of follow-up, bone metastasis developed in 48 patients (11.40%); while 373 patients (88.60%) remained metastasis-free. All patients were randomly allocated to a training cohort (*n* = 317) and a validation cohort (*n* = 104) in a 75%:25% ratio using complete randomization implemented in R software. Baseline data comparison is presented in Table 1.

As presented in Table 1, the primary tumor location was left-sided in 53.31% of cases and right-sided in 46.69%. The most frequent site was the upper outer quadrant of the breast (52.68%). Luminal A subtype (HR+/HER2−) was the predominant molecular subtype, accounting for 62.46% of cases. The majority of patients exhibited positive estrogen receptor (ER; 75.39%) and progesterone receptor (PR; 67.82%) status. The most common T stage and N stage were T1 (58.04%) and N0 (58.05%), respectively. Given that prior literature reports and our preliminary analysis suggested a weak correlation between the composite AJCC TNM stage and bone metastasis risk, T stage and N stage were included as separate variables in this analysis to allow a deeper investigation into the influence of different aspects of tumor burden. Invasive ductal carcinoma represented 91.17% of breast cancer pathological types.

### 3.2 LASSO regression analysis summary

**Figure 2:**
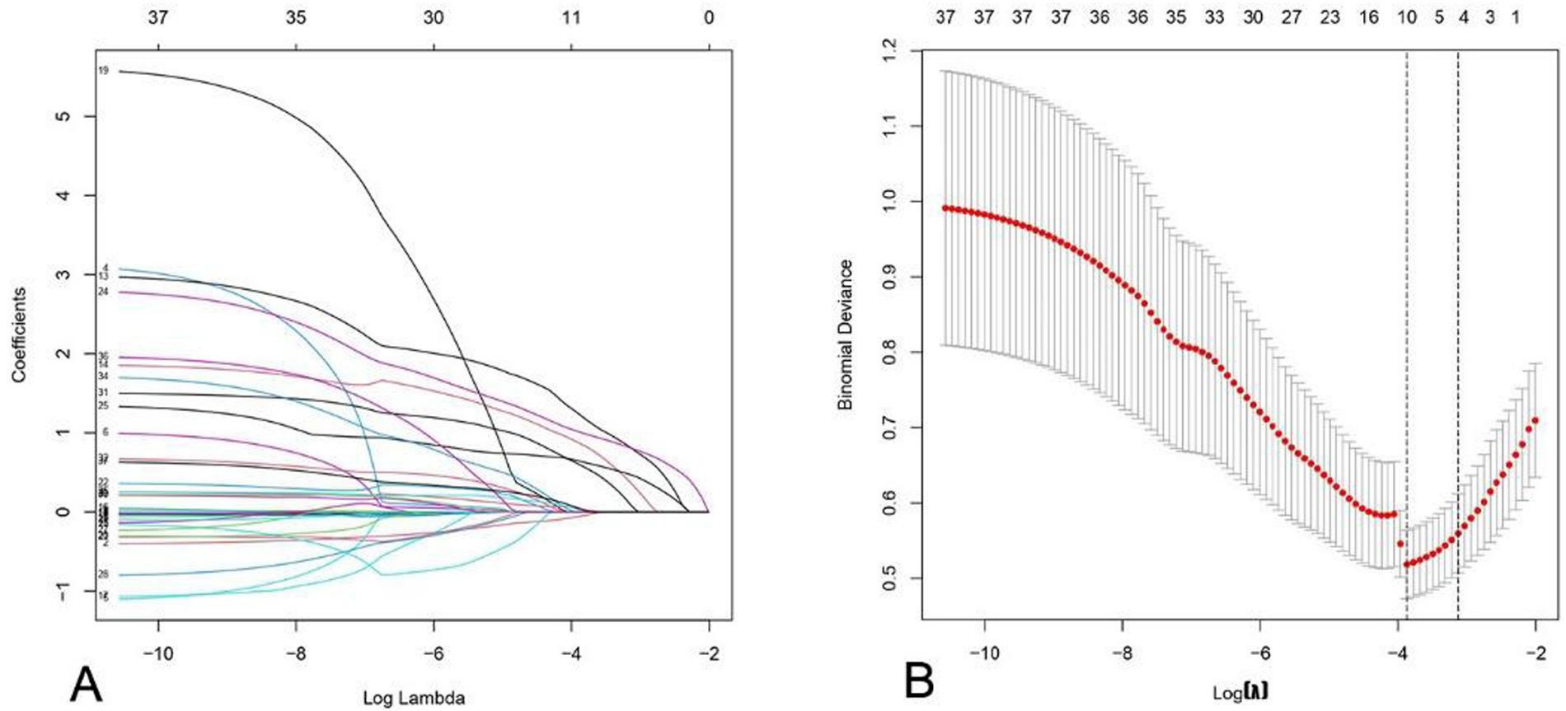
Characteristics of coefficient variation in LASSO regression and analysis of binomial deviance plots. **(A)** In LASSO regression analysis, as the penalty coefficient increases, ultimately resulting in all coefficients being reduced to zero, with the aim of eliminating irrelevant variables. **(B)** The position at the left dashed line indicates the point of minimal error, where the model exhibits optimal performance.

We performed LASSO regression on the training cohort data (Figures 2A,B). Figure 2A demonstrates that as the penalty coefficient increased, coefficients of all independent variables shrank towards zero, ultimately being reduced to zero. Figure 2B indicates that the binomial deviance reached its minimum when the predictive model incorporated 10 variables. Features with non-zero coefficients selected through this process included: age at menopause, CA125 level, neoadjuvant chemotherapy, family history of cancer, non-osseous distant metastasis, axillary lymph node metastasis (ALNM), T stage, marital status, primary tumor location, and blood type.

### 3.3 Construction of the nomogram model

**Figure 3:**
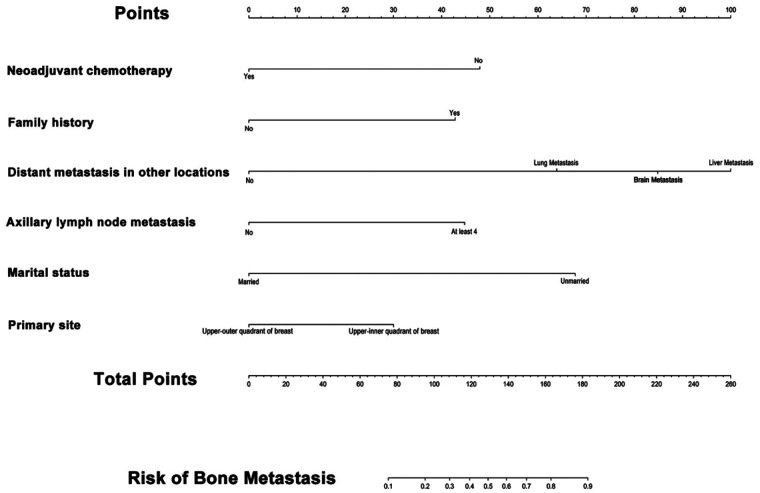
The nomogram predictive model for BMBC. For instance, a patient who failed to receive the recommended neoadjuvant chemotherapy (NAC) (score: 47.5 points), presented with a family history of cancer (score: 42.5 points), exhibited no distant metastasis (DM) (score: 0 points), showed absence of axillary lymph node metastasis (ALNM) (score: 0 points), maintained married status (score: 0 points), and had a primary lesion located in the upper-inner quadrant of breast (score: 30 points), the cumulative risk score totals approximately 120 points. According to the nomogram analysis, this score corresponds to a 40% probability of developing bone metastasis from breast cancer within 5 years, as calibrated by the probabilit*y* axis on the lower horizontal scale.

Variables exhibiting non-zero coefficients in the least absolute shrinkage and selection operator (LASSO) regression analysis were selected as candidates. This process refined the initial predictor set to 10 features by applying a penalty (lambda) to shrink coefficients and prevent overfitting. These 10 variables were subsequently entered into a multivariable logistic regression model to ascertain independent predictors of breast cancer bone metastasis. The results of the multivariable logistic regression are presented in Table 2. This analysis revealed that six variables retained statistical significance and emerged as independent predictors: neoadjuvant chemotherapy, family history of cancer, non-osseous distant metastasis, axillary lymph node metastasis (ALNM), marital status, and primary tumor location. The regression coefficients (β), odds ratios (OR), and corresponding confidence intervals for each of these predictors are detailed in Table 2. Based on these results, a predictive nomogram was constructed to provide a visual and quantitative tool for individualized risk assessment (Figure 3).

**Table 2:** Non-zero coefficient variables in multifactorial logistic regression analysis results.

Fators	Regression coefficient	OR (95% CI)	*p* value
Intercept	0.332	1.39 (0.01–278.28)	0.902
Age of menarche	−0.282	0.75 (0.55–1.03)	0.074
CA125	−0.007	0.99 (0.96–1.02)	0.644
Non-Neoadjuvant Chemotherapy	2.591	13.35 (3.19–55.92)	<0.01
Family history of cancer	1.635	5.13 (1.82–14.43)	<0.01
Distant metastasis in other locations			
No other distant transfer	1.00 (reference)		
Lung Metastasis	2.865	17.55 (3.59–85.74)	<0.01
Liver Metastasis	4.730	113.24 (8.89–1,441.72)	<0.01
Brain Metastasis	3.787	44.14 (5.64–345.38)	<0.01
ALNM			
No axillary lymph node metastasis	1.00 (reference)		
Less than 4	0.839	2.31 (0.51–10.60)	0.280
At least 4	2.156	8.64 (2.09–35.75)	<0.01
T Stage			
T1	1.00 (reference)		
T2	0.239	1.27 (0.41–3.93)	0.678
T3	0.868	2.38 (0.28–20.62)	0.430
Marital status			
Unmarried	1.00 (reference)		
Married	−2.716	0.07 (0.01–0.87)	0.039
Widowed	−0.265	0.77 (0.04–16.14)	0.864
Divorced	−1.680	0.19 (0.00–17.52)	0.469
Primary site			
Upper-outer quadrant of breast	1.00 (reference)		
Lower-outer quadrant of breast	0.908	2.48 (0.48–12.72)	0.276
Upper-inner quadrant of breast	1.509	4.52 (1.08–18.99)	0.039
Lower-inner quadrant of breast	1.022	2.78 (0.55–14.10)	0.218
Accumulated nipple	−13.003	2.25 × 10⁻^6^ (0–∞)	0.994
Blood type			
O	1.00 (reference)		
A	1.191	3.29 (0.68–15.84)	0.137
B	1.302	3.68 (0.85–15.92)	0.082
AB	1.192	3.29 (0.59–18.30)	0.173

The nomogram graphically represents the multivariable logistic regression model. Each of the six independent predictors is assigned a separate axis (“Points” scale). The length of each axis is proportional to the contribution (regression coefficient β) of that variable to the outcome. To use the nomogram, the value for a given patient for each predictor is located on its respective axis, and a vertical line is drawn upward to the ‘Points' line to determine the corresponding score.

For example, a patient with a family history of cancer would receive a higher point contribution than one without, reflecting its positive association with bone metastasis risk. Conversely, married marital status contributes fewer points, aligning with its role as a protective factor. The points for all six variables are then summed to obtain a total points score. This total is subsequently located on the “Total Points” axis. Finally, by drawing a line straight down from the total points to the “Risk of Bone Metastasis” axis, the clinician can read the patient's estimated probability of developing bone metastasis, ranging from 0 to 1.

This nomogram effectively translates the complex statistical model into an intuitive, user-friendly scoring system. It allows for the integration of continuous and categorical variables to generate an individualized risk prediction, facilitating immediate clinical interpretation and supporting shared decision-making at the point of care, without the need for complex calculations.

### 3.4 Validation of the nomogram

**Figure 4:**
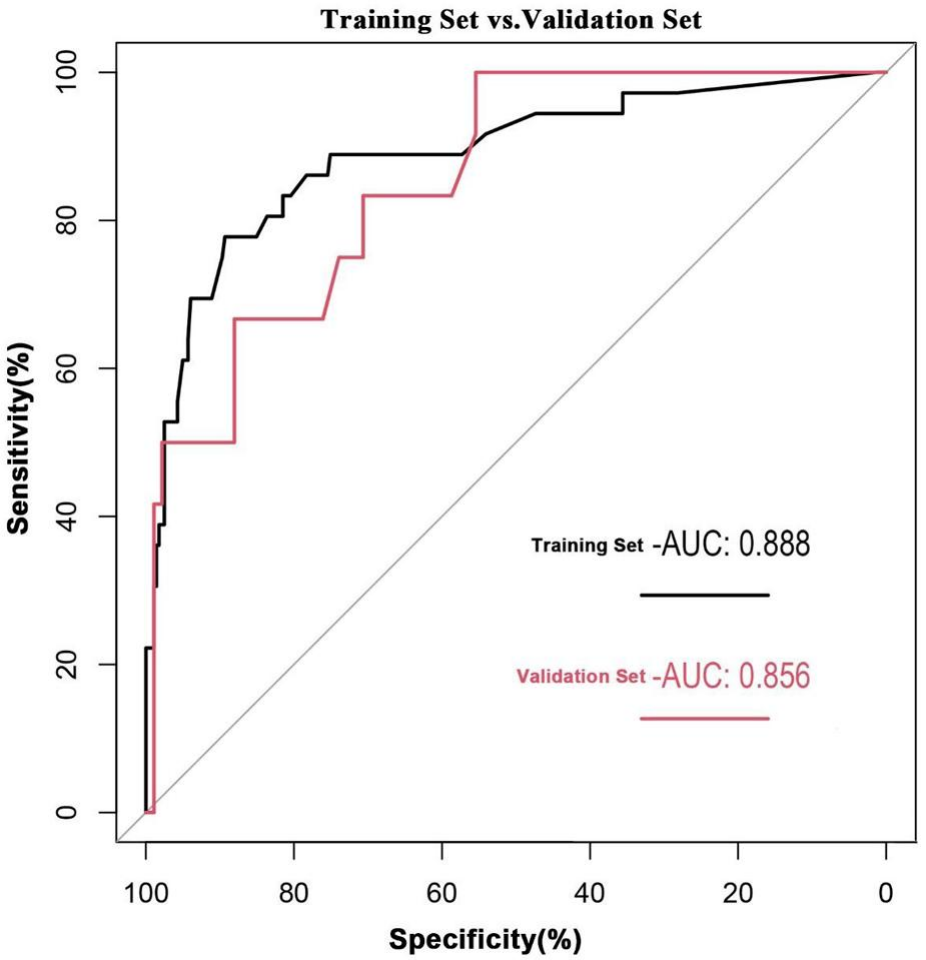
Receiver operating characteristic curve of the nomogram prediction model.

**Figure 5:**
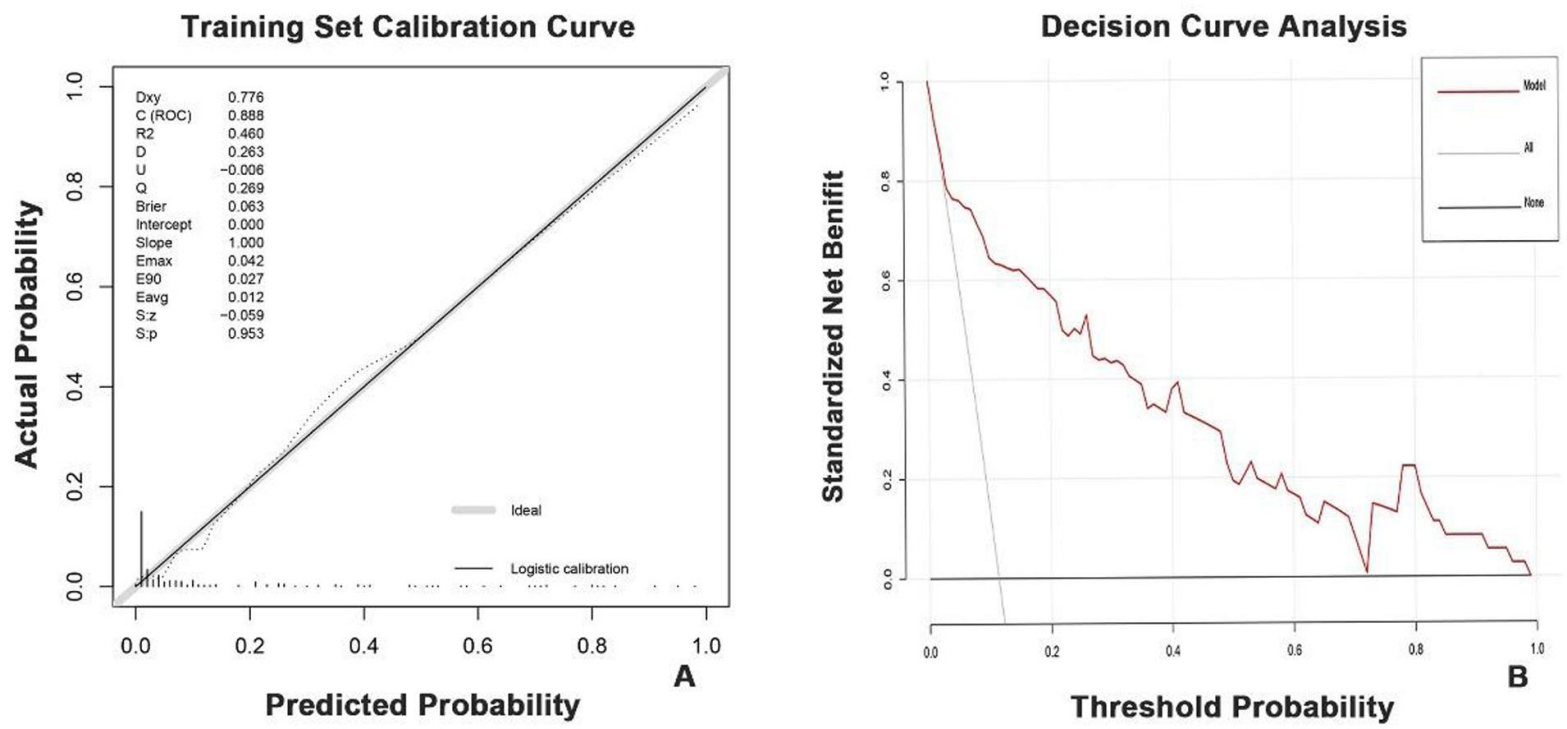
Calibration curve and Decision curve analysis for the nomogram prediction model. **(A)** Calibration curve for the training set data. The closer the black line is to the diagonal gray line, the higher the accuracy of the predictive model. **(B)** Decision curve analysis for the nomogram prediction model. The red line represents the nomogram model constructed in this study. The higher the position of this curve, the greater the net benefit of the model in predicting bone metastasis. The gray line represents the strategy of intervening in all patients (assuming all patients will develop bone metastasis), while the black line represents the strategy of not intervening in any patients (assuming no patients will develop bone metastasis). The decision curve analysis of this predictive model demonstrates that across the bone metastasis risk threshold range of 0 to 1, using this model

Both the training and validation cohorts achieved high AUC values (0.89 and 0.86, respectively), demonstrating excellent model discriminative performance (Figure 4). As shown in Figure 5A, the *x*-axis represents the predicted probability of breast cancer bone metastasis, while the *y*-axis denotes its actual occurrence. Predictive accuracy increased with closer alignment of the calibration curve to the diagonal reference line. Hosmer- Lemeshow tests indicated good calibration in both cohorts (training cohort *p* = 0.7436; validation cohort *p* = 0.1142). Both *p*-values exceeded 0.05, indicating no significant deviation between predicted and observed outcomes and supporting good model fit. Comprehensive evaluation incorporating multiple assessment metrics demonstrated consistently high performance of the developed predictive model in the training and validation cohorts.

Decision curve analysis (DCA) of the prediction model (Figure 5B) demonstrated added net benefit across the entire risk threshold spectrum (0–1) for breast cancer bone metastasis, outperforming both “treat-all” and “treat-none” strategies. This indicates broad clinical applicability of the nomogram.

## 4 Discussion

Metastasis represents the primary cause of mortality in the majority of breast cancer patients (25). Bone metastasis imposes a dual burden characterized by its high incidence rate and propensity for severe complications (26). Critically, upon diagnosis of bone metastasis, patients experience significantly compromised survival outcomes, with median survival further reduced to 2.1–4 years (5). Although current imaging modalities (e.g., bone scintigraphy, PET/CT) can detect osseous metastases, their application faces significant limitations. Traditional imaging screening demonstrates low detection probability and high false-positive rates for most patients with low metastatic risk—particularly stages I-II breast cancer. Moreover, these approaches impose substantial financial burdens and heighten patient anxiety regarding ionizing radiation exposure (27, 28). However, no readily applicable prediction model currently exists that simultaneously graphically represents both risk and protective factors for delayed bone metastasis in newly diagnosed breast cancer patients.

Therefore, the development of a practical risk prediction model to identify breast cancer patients at high risk of bone metastasis and facilitate personalized interventions holds significant clinical value. The nomogram constructed in this study integrates six independent predictors—neoadjuvant chemotherapy, family history of cancer, non-osseous distant metastasis, axillary lymph node metastasis, marital status, and primary tumor location—to translate a multivariate logistic regression model into an intuitive graphical scoring system. Unlike conventional risk stratification methods, the nomogram enables clinicians to rapidly and quantitatively assess individualized risk for each patient, thereby avoiding a one-size-fits-all approach. For instance, for a married patient with no family history of cancer but with ≥4 metastatic axillary lymph nodes, the nomogram provides a precise probability of risk through cumulative points, rather than simplistically classifying the case as “high-risk” or “low-risk.” Such granular risk quantification supports the shift from population-based medicine to personalized care, aligning with the principles of precision medicine (18, 29).

Although some popular machine learning models are capable of performing such predictive tasks, their clinical utility is often limited by the “black-box” nature of complex algorithms, which compromises interpretability (17). In contrast, the decision-making logic of the nomogram developed in this study is fully transparent: the contribution of each variable is clearly reflected through axis length and point assignment, making it easy for both physicians and patients to understand the sources of risk. This transparency greatly facilitates shared decision-making between clinicians and patients, enabling more informed participation in treatment choices and improving adherence. In clinical practice, this nomogram offers a practical tool for long-term management of breast cancer patients. On one hand, for patients predicted to be at high risk (e.g., those with a bone metastasis probability >20%), clinicians may recommend intensified follow-up strategies—such as shortening the interval for bone scintigraphy or PET-CT scans—and consider prophylactic use of bisphosphonates to reduce the risk of skeletal-related events. On the other hand, for low-risk patients (e.g., those with a probability <5%), unnecessary routine bone scans can be avoided, thereby reducing medical costs, radiation exposure, and patient anxiety.

In this study, we analyzed 421 patients with 5-year follow-up data and identified six independent predictors of bone metastasis in breast cancer. Consistent with prior reports, ≥4 metastatic axillary lymph nodes constituted an independent risk factor for bone metastasis. Marco Colleoni et al. demonstrated that patients with ≥4 involved axillary lymph nodes at diagnosis exhibited the highest cumulative incidence of bone metastasis (30). Similarly, Chen Zhao et al. reported a significant correlation between higher numbers of metastatic axillary lymph nodes and breast cancer bone metastasis (16). Yann Delpech et al. further confirmed that nodal status was independently and significantly associated with subsequent bone metastasis in breast cancer. Our findings align with these studies, showing that patients with ≥4 metastatic axillary lymph nodes had significantly higher likelihood of developing bone metastasis compared to those without nodal involvement.

Notably, marital status was identified as an independent predictor of bone metastasis in breast cancer, with married status serving as a protective factor. This finding aligns with previous reports: Gao et al. identified unmarried status as a risk factor for bone metastasis in breast cancer patients (31), while Guo et al. demonstrated a positive correlation between unmarried status and both the incidence and poor prognosis of bone metastasis (32) Furthermore, in the risk prediction model developed by Li et al., non-married marital status was significantly associated with increased bone metastasis risk (33). Collectively, these studies suggest that unmarried women face higher risks of bone metastasis post-diagnosis and exhibit poorer prognosis compared to their married counterparts—a conclusion consistent with our findings.

The association between marital status and bone metastasis risk may be underpinned by several interlinked pathophysiological and psychosocial mechanisms. First, from a biological perspective, reproductive history and endogenous hormone exposure differ by marital status. Unmarried/nulliparous women may experience longer periods of uninterrupted ovulation and cumulative estrogen exposure, a known promoter of breast cancer progression. Evidence suggests that estrogen can upregulate factors like parathyroid hormone-related protein (PTHrP) and receptor activator of nuclear factor kappa-B ligand (RANKL) within the bone microenvironment, thereby facilitating the colonization and growth of metastatic cells (34, 35). Second, chronic stress associated with social isolation or lack of spousal support may dysregulate the hypothalamic-pituitary-adrenal (HPA) axis, leading to elevated cortisol levels and systemic inflammation. This pro-inflammatory state, characterized by increased levels of cytokines such as IL-6 and TNF-α, can promote tumor cell invasion, angiogenesis, and bone resorption, creating a favorable niche for metastasis (36). Third, differences in healthcare-seeking behaviors and treatment adherence are well-documented. Married patients often benefit from spousal encouragement and logistical support, leading to better compliance with adjuvant therapies, timely follow-up, and earlier management of complications—all critical for metastasis suppression (37, 38). Our results underscore the need for heightened clinical vigilance regarding treatment adherence and follow-up compliance in unmarried breast cancer patients. Enhanced awareness and tailored interventions for this demographic may improve outcomes.

Additionally, this study identified distant metastasis in other locations as an independent risk factor for bone metastasis. These include lung, brain, and liver metastases. Metastasis to one organ may accelerate cancer spread to others. Bone metastasis could substantially increase risks of additional metastases elsewhere, and vice versa (39). Prior studies confirm our findings: Brain, lung, and liver metastases significantly correlate with skeletal metastasis risk (8, 33). Thus, patients with existing non-bone metastases require enhanced surveillance for bone metastasis risk and timely implementation of targeted interventions to prevent further deterioration in quality of life.

Concurrently, our study identified completion of guideline-recommended neoadjuvant chemotherapy as an independent protective factor against bone metastasis in breast cancer. Current research primarily focuses on neoadjuvant chemotherapy's role in tumor shrinkage and surgical outcomes (40). Emerging evidence confirms its protective effect: Preoperative neoadjuvant chemotherapy improves surgical success and may reduce bone metastasis risk (41–43). Mechanistically, neoadjuvant chemotherapy directly eliminates disseminated tumor cells in bone marrow niches. It particularly targets EpCAM-positive subpopulations, suppressing metastatic seeding (44). Through such actions—including micro-metastasis eradication—it confers significant protection against skeletal spread. This study pioneers the incorporation of this biological mechanism into bone metastasis prediction models. Our approach enhances clinical utility. For guideline-eligible patients, strict adherence to complete neoadjuvant chemotherapy regimens must be enforced to mitigate subsequent metastasis risk, with mandatory therapeutic response monitoring.

Studies indicate higher rates of sternal bone metastasis in patients with inner-quadrant primary tumors. This likely reflects medial breast tissue's lymphatic drainage toward parasternal nodes (45). Our study confirms Upper-inner quadrant tumor location is an independent risk factor for breast cancer bone metastasis. Its higher regression coefficient warrants enhanced vigilance: Prioritize rigorous imaging review for inner-quadrant primary tumors.

Risk factor analysis identified neoadjuvant chemotherapy, family history of cancer, distant metastasis to other sites, axillary lymph node metastasis, marital status, and primary tumor location as factors associated with bone metastasis in breast cancer patients. Notably, the baseline table comprehensively lists all parameters collected in this study, which were selected based on an extensive literature review of factors related to breast cancer bone metastasis. The systematic collection and presentation of these parameters provide a more complete informational background for clinical risk assessment. Although some parameters showed that these factors may have limited direct predictive value for bone metastasis in the specific population or context of this study, or their effects might be overshadowed by stronger predictors, they still hold significant clinical relevance. For instance, although breast cancer subtype was not included as a core predictive variable, it is important to note that breast cancer subtype is an extremely important biological parameter in assessing the risk of bone metastasis. Previous studies have confirmed that different subtypes exhibit significant heterogeneity in distant metastasis tendencies and sites due to their intrinsic molecular characteristics and tumor microenvironment differences (46). Therefore, we still recommend that clinicians consider the predictive model established in this study in conjunction with important biological characteristics such as breast cancer subtype when evaluating individual patient risk, to form a comprehensive and multidimensional judgment. Future studies could further explore the potential value or interactions of these factors in larger sample sizes.

This study offers several notable advantages: First, the model was constructed using clinical data from a Northern Chinese breast cancer cohort, providing a more accurate reflection of the biological characteristics and bone metastasis-related information specific to this regional population. Second, compared to prior bioinformatics studies reliant on public databases (e.g., SEER), our analysis incorporated more comprehensive variables, including detailed records of systemic therapies such as neoadjuvant chemotherapy regimens, endocrine therapy, and targeted agent administration. This granular classification of treatment information addresses a significant gap in previous public database studies, enabling the provision of precise guidance for individualized treatment strategies (8). Third, models developed for specific populations may exhibit limited generalizability to others. By utilizing local clinical data, the present model is likely to demonstrate greater compatibility with Chinese patients than prediction models derived from public databases primarily representing European and American populations.

However, this study has several limitations. Although the model demonstrated excellent predictive performance, as indicated by the ROC curves and calibration plots, its single-center, retrospective design inevitably led to the exclusion of some cases and may have introduced selection bias. Furthermore, the lack of external multi-center validation necessitates future multi-center and prospective studies to enhance the generalizability of our findings. We plan to expand data collection in subsequent research to further improve the model's reliability.

## 5 Conclusions

Our nomogram demonstrates that failure to receive appropriate neoadjuvant chemotherapy, a family history of cancer, the presence of distant metastases in other locations, involvement of four or more metastatic axillary lymph nodes, unmarried status, and primary tumor location are independent risk factors for bone metastasis in breast cancer patients. By constructing the nomogram, we aimed to identify high-risk patients with potential bone metastasis, thereby enhancing predictive accuracy for this outcome. The model exhibited robust predictive performance and reliability during both internal evaluation and external validation. Early risk stratification using this tool may optimize follow-up and therapeutic strategies, assisting clinicians in formulating personalized precision medicine approaches.

## Data Availability

The original contributions presented in the study are included in the article/Supplementary Material, further inquiries can be directed to the corresponding author.
